# Diet, Microbiota and Memory: How What We Eat Affects Memory and Cognitive Processes and Whether Diet Can Help Prevent the Onset of Dementia

**DOI:** 10.7759/cureus.115384

**Published:** 2026-08-28

**Authors:** Milena Sucharska, Karolina Gardas, Karolina Hernik, Artur Brudek, Jan Pawlasek, Hubert Piwar, Monika Lis, Mateusz Sucharski, Stanislaw Kisiel, Patryk Cierpial, Pawel Mikolajczak, Magdalena Puziuk, Katarzyna Kupczyk, Zofia Jedra

**Affiliations:** 1 Medicine, University of Warsaw, Warsaw, POL; 2 Pharmacotherapy and Pharmaceutical Care, Medical University of Warsaw, Warsaw, POL; 3 Medicine, Jan Kochanowski University, Kielce, POL; 4 General Practice, Dr. Anna Gostynska Wolski Hospital, Warsaw, POL; 5 General, Vascular and Oncological Surgery, Mazovian Hospital Bródnowski, Warsaw, POL; 6 General Practice, Military Institute of Medicine - National Research Institute, Warsaw, POL; 7 Medicine, Central Clinical Hospital in Warsaw, Warsaw, POL; 8 Medicine, National Medical Institute of the Ministry of the Interior and Administration, Warsaw, POL; 9 Medicine, Dr. Aleksander Majkowski District Hospital, Kartuzy, POL; 10 General Medicine, Warszawski Szpital Południowy, Warsaw, POL; 11 General Medicine, Professor W. Orłowski Independent Public Clinical Hospital, Warsaw, POL

**Keywords:** cognitive function, dementia, diet, memory, microbiota

## Abstract

This paper was written in response to the growing interest in the influence of the gut-brain axis on the development of dementia and cognitive disorders. The main aim was to review and summarise the latest findings in this area. The article was divided into a section on animal studies, which sought to link specific macronutrient ratios in diets to general behavioural disturbances in organisms, alongside the development of hypotheses regarding changes in the levels of substances responsible for, or indicative of, neurodegenerative processes. Subsequently, the impact of diets containing carbohydrates, proteins, and fats on cognitive processes and memory in humans was discussed. Deterioration was observed particularly among groups consuming large amounts of fat and experiencing a caloric surplus, which led to the development of obesity. Finally, the study presented both dietary interventions and multifactorial lifestyle changes aimed at improving memory function in patients, including older adults and those with Alzheimer’s disease. A particularly significant factor highlighted in most studies was adherence to the Mediterranean and Mediterranean-DASH Intervention for Neurodegenerative Delay (MIND) diets, which most likely contributed to slowing the progression of dementia in the majority of patients. Several studies have demonstrated a link between the qualitative composition of the diet and the development of dementia. Obesity, resulting from a high intake of fat and/or carbohydrates, and the inflammation it provokes, including within the central nervous system, plays a key role in this process. The most beneficial effects were demonstrated by the MIND and Mediterranean diets when combined with comprehensive lifestyle changes, which is why the authors were cautious about drawing definitive conclusions.

## Introduction and background

In recent years, increasing attention has been paid to the impact of a healthy digestive system, with particular emphasis on the composition and biodiversity of the gut microbiota, on the functioning of individual organs in the human body [1-4]. One area of interest for researchers is the effect of dietary intervention on memory decline and its potential slowing [5-11]. To this end, clinical studies have been undertaken to demonstrate the broad impact of potential non-pharmacological interventions, alongside laboratory studies involving experimental animals that attempt to objectively assess the results obtained or aim to establish links between specific molecules present in the central nervous system (CNS) and cognitive functions [12-18].

The main issues discussed by researchers included the proportions of macronutrients [19-27], with a focus on the impact of an excess of one of them on cognitive processes, as well as the application of widely accepted dietary patterns, such as the Mediterranean diet or the MIND (Mediterranean-DASH Intervention for Neurodegenerative Delay) diet, which focuses on 10 brain healthy food groups (green leafy vegetables, other vegetables, nuts, berries, beans, whole grains, seafood, poultry, olive oil and wine) and traditionally has a positive effect on memory, particularly in older people [28-33]. This review aims to discuss recent literature on the main topic, along with previous studies, summarise the findings, and formulate brief recommendations for implementation in older patients at risk of developing dementia.

## Review

Materials and methods

On 15th April 2026, the authors reviewed the latest publications in databases such as PubMed, Google Scholar, Cochrane Library and Scopus. Studies were included regardless of the journal of publication, provided they were in English or had an official English translation available. The search was divided into 3 groups: animal studies, macronutrients, or interventions. The databases were searched using the same keywords. Simultaneously, the inclusion criteria were keywords used in the groups (accordingly: 'diet, memory loss, cognitive function, mice, microbiota, intervention, macronutrients') and then were excluded if the study did not match the aimed topic (for example: interventional studies with use of pharmaceutics instead of diet changes, animal experiments not based on changes in their diet, etc.). In the group including articles on macronutrients, 109 records were found; 94 abstracts were screened, 49 were assessed in full, and nine qualified for review. In the group involving animal studies, 422 records were found; 322 abstracts were screened, 87 were assessed in full, and nine qualified for review. In the case of interventional studies, 300 records were found, 198 abstracts were screened, 75 were assessed in full, and 17 qualified for review. In addition, three more articles were cited on a meritorious basis, filling in information lacking in the reviewed studies, and one article was used as a key source for creating recommendations. All four additional texts were picked manually.

Results

Animal Studies

As indicated in the introduction, the topic of the possible links between diet and changes in the gut microbiota and the overall functioning of the nervous system has been addressed by researchers since the 1960s with increasing interest. Laboratory experiments using mice [1,2,12-17] allowed verification of chronologically emerging hypotheses regarding specific mechanisms regulating cognitive functions, as well as the conduct of prospective studies on the possible influence of diet on the development of dementia [12,14]. The studies [12-17] described the effect of specific changes in the macronutrient composition of mouse feed on the animals’ subsequent cognitive functioning and memory. Depending on the experimental design adopted, the mice were fed a standard rodent diet from birth and then assigned to experimental or control groups. The start of the intervention ranged from 10 to 18 weeks of age. Following a few weeks of feeding, the animals were subjected to tests (Y-maze test, Water-maze test, circular platform test and New Object Recognition test). The authors [12-17] then euthanised the mice, collected brain tissue, with particular focus on the hippocampus [12,14,15], or without [13,17], and conducted chemical and/or immunohistochemical analyses. The results obtained allowed for the conclusion that a high-fat and high-carbohydrate diet has a negative impact on memory.

In the case of an increased carbohydrate intake, Trevino et al. [17] pointed out that an imbalance in sugar intake promoted significant fat accumulation, the excess of which (also demonstrated in the study) is a recognised pro-inflammatory factor [34]. In samples taken from the temporal cortex and hippocampus, there were on average approximately 30 astrocytes, identified immunohistochemically using glial fibrillary acidic protein (GFAP), compared with approximately 4.5 cells of this type in the control sample. The numbers of cells were counted on a field of 500 µm². Furthermore, the chemical composition of the brain tissue homogenate revealed concentrations of interleukin 1-β that were 163% and 275% higher, respectively, from values 28.0 +/- 2.2 and 13.6 +/- 1.8 pg/ mg of protein, to 73.8 +/- 5.0 and 51.1 +/- 741 pg/ mg of protein in high-calorie diet group, and tumour necrosis factor (TNF)-alpha levels that were 224% and 108% higher, measurements for the cerebral cortex and hippocampus, respectively, from values 15.9 +/- 2.0 and 16.4 +/- 13.9 pg/ mg of protein, to 48.7 +/- 6.8 and 29.0 +/- 7.0 pg/ mg of protein in high-calorie diet group. At the same time, lipid peroxidation markers were increased by 116% and 96% compared to the control group, respectively, from values 0.66 +/- 0.07 and 0.61 +/- 0.08 URF/ mg of protein, to 1.43 +/- 0.13 and 1.18 +/- 0.25 URF/ mg of protein in high-calorie diet group. Functionally, this translated into the results of the animals’ spatial memory tests, e.g., it took them longer to find the correct platform, they were approximately 24% less active compared to the control group (activity measured by counting entries onto the target platform) and, after 10 days of training, the time taken by mice from the experimental group to locate the correct platform again was 36% longer than in the control group.

The link between obesity and impaired cognitive function was also observed in another study [19] discussed later; however, among the available animal models, the experiment by N’Diaye et al. [18] demonstrated impaired memory and anxiety-like responses in male mice following a 15-week period of feeding a high-carbohydrate, high-fat diet. Interestingly, this effect did not apply to female mice. At this point, it is worth mentioning the potential protective effect of oestradiol on cognitive function and neuronal plasticity when following a high-fat diet, which was investigated by Felintro et al. [13]. Mice with an average age of approximately 9.5 months were divided into two groups and fed a high-fat or low-fat diet for 12 weeks, after which they underwent bilateral ovariectomy. The diet was continued, but the experimental group received oestradiol supplementation. After a further 40 days, the researchers began behavioural testing using the New Object Location test. At the same time, it was found that the experimental group gained less body weight compared to the control group (an average gain 18% lower). No such differences were observed in the group fed a low-fat diet, regardless of oestradiol administration. The training phase revealed no significant differences between the groups. In memory tests, animals supplemented with oestradiol achieved better results regardless of the type of diet used, with the group fed a low-fat diet achieving an additional 36% better results compared to the high-fat group. These latter results could indicate a significant influence of diet, particularly in the group of older animals.

Hence, the next question that arises is whether there is a link between the duration of a particular diet and cognitive function in an animal model. Costa et al. [15], building on earlier work, focused their analysis on the conditioning of memory consolidation in the hippocampus by cholinergic neurons. Four-week-old male mice were divided into two groups (experimental and control), with the key difference being the inclusion of a high-fat diet. Additionally, in some mice, the link between cholinergic pathways and hippocampal processes was confirmed using injections of α7 nicotinic acetylcholine receptor analogues. The diet was introduced in the experimental group for a period of three days, which did not result in significant changes in body weight between the groups; however, an increase in calorie intake of approximately 12% was observed in the high-fat diet group. Behavioural tests were then conducted using the Open Field test and the Novel Object Recognition test. In the open field test, it was shown that mice from the high-fat diet group were less likely to adopt a two-legged stance; apart from this, no significant differences in behaviour were reported. In the novel object recognition test, however, mice from the experimental group took approximately 1.5 times longer to complete the task compared to the control group. In preparations subsequently made from the hippocampi of the tested mice, approximately 50% lower expression of α7nAChR receptors was observed. Levels of the cytokines and chemokines tested remained comparable in both groups; however, increased phosphorylation was observed in the Signal Transducer and Activator of Transcription 3 (STAT3), c-Jun N-terminal Kinase (JNK), IκB Kinase (I-kappa-B Kinase; IKK) pathways, which are indirectly responsible for transmitting information about ongoing inflammation and serve to activate lymphocytes, suggesting a developing inflammatory response.

In summary, even short-term consumption of increased amounts of fat may influence the development of CNS inflammation and impair memory function in animal models. The authors of the above studies used diets with a broad lipid profile in their experiments. The results obtained indicated a generally negative effect of a high lipid intake.

A final point of interest in the field of laboratory research is the effect of diet on changes in the gut microbiota that influence memory and behavioural functioning in laboratory animals. Wang et al. [2] conducted an experiment in which a feed mixture with a higher beef protein content was used. Analysis of the animals’ faeces showed that the experimental group exhibited greater diversity of gut flora, as demonstrated by the presence of species-specific DNA in polymerase chain reaction (PCR) tests. A ‘hole board apparatus’ was used to analyse the mice’s behaviour, assessing a range of parameters including memory, exploration time and anxiety-related behaviours. From the results obtained, it was concluded that the group on the protein-rich diet demonstrated statistically significantly greater mobility, better memory and lower levels of anxiety compared to the control group. Samulenaite et al. [1], in turn, conducted a more detailed study involving the collection of faecal samples from human donors, whilst dividing them into groups based on their cognitive performance scores in the Stroop test. Following prior antibiotic treatment, faecal microbiota transplantation (FMT) was performed on mice, which were then divided into groups fed either a standard diet or a high-fat diet whilst receiving the medication. Behavioural tests were conducted between each intervention. The results showed that the inclusion of antibiotic therapy did not induce changes in animals on a high-fat diet, but it caused a significant impairment of short-term memory, which was subsequently reversed by microbiota transplantation from donors with a low Stroop score and improved with flora from donors with an optimal test score. In the case of long-term memory, FMT reversed memory impairment in the high-Stroop-score diet group and in the high-fat diet group with a low Stroop score. Working memory and depressive-anxiety behaviours in the mice showed no differences between the groups at any stage of the experiment.

In summary, the presented studies demonstrated, under laboratory conditions, links between the use of specific types of diets, particularly a high-fat diet, and the deterioration of cognitive function and memory. At the same time, it was demonstrated that there is a correlation between these parameters and changes in the composition of the gut microbiota, both induced by the type of food consumed [2] and independently of it [1].

Classification Based on Macronutrients and Their Potential Impact on Function

The macronutrients contained in food, i.e. carbohydrates, proteins and fats, provide humans with the nutrients essential for survival. They influence normal brain growth and development in children, as well as memory and all cognitive processes. The role of individual components in shaping well-being, and in preventing or slowing the progression of many diseases, including cardiovascular and neurodegenerative conditions, as well as depression, is also highlighted [20]. Both a deficiency and an excess of specific macronutrients can negatively affect the body’s functioning.

Recently, the topic of sugar’s impact on brain function has become highly significant and frequently discussed, partly due to the lifestyle of modern society, increased sugar consumption and more frequent neurological problems [21,35]. Muth et al. [20] highlighted the negative impact of simple sugars on all cognitive functions of the body, such as working memory, attention and visuospatial processing. These results were obtained using cognitive tests, i.e., the word list learning test, the digit span test, the clock drawing test, the figure copying test, the Stroop test and the verbal fluency test. One of these studies [20] showed that a daily sugar intake of 60 g was associated with a decline in the Mini Mental State Examination (MMSE) score, corresponding to a level typical of people approximately 10 years older. On the other hand, the effect of higher fibre intake on cognitive functions was also investigated. Interestingly, the results showed a beneficial effect of a higher fibre intake on the entire process of brain ageing, including improved cognitive function and a reduced risk of chronic diseases.

In a study on the impact of nutrients and brain metabolism, Oleson et al. [19] noted that the consumption of carbohydrates or proteins does not significantly affect neuronal mortality, but has a major impact on the development of obesity and metabolic syndromes, which contribute to a significant deterioration in cognitive brain function. Given the above information, the impact of a low-carbohydrate diet on cognitive processes becomes an interesting issue. Charbit et al. [5] linked adherence to a low-carbohydrate diet with improved memory function, which was confirmed by memory tests in older adults. This may also be due to the increased levels of ketones in a low-carbohydrate diet, which have positive effects on the process of recalling information. Contrasting observations were reported in the study by Arsalandeh et al. [6], whose findings show that, over a longer study period, a low-carbohydrate diet contributes to increased anxiety and slows down thinking processes in people over 55 years of age. Opinions on the appropriate carbohydrate content in the diet are divided. The above studies suggest that a high intake of sugars has a negative impact on cognitive function. However, it should be remembered that excessive restriction of carbohydrate intake may also have adverse effects. 

Another extremely important macronutrient in the diet is protein. The amino acids released during protein digestion are essential for the synthesis of neurotransmitters, which ensure the proper functioning of the brain and nervous system, and are thus responsible for learning, memory and concentration. This was confirmed by the results of several studies [22-24], which demonstrated a significant and positive association between a high-protein diet and memory processes. A particular improvement was noted in visuospatial abilities, verbal fluency, as well as processing speed and attention span.

Keum et al. [23] further concluded that the positive effect of protein on episodic memory and all cognitive functions may be linked to apolipoprotein E4 (APOE4), which acts as a moderator of this relationship. APOE4 is the main genetic risk factor for the development of late-onset Alzheimer’s disease (AD) and atherosclerosis. Hence, they concluded that a high-protein diet should be particularly beneficial in maintaining good episodic memory in individuals predisposed to developing AD. Furthermore, in the study by Li et al. [24], in which the study group consisted of people over 60 years of age, it was shown that higher intake of protein from milk and dairy products was associated with poorer cognitive function. The authors observed this relationship by conducting a series of cognitive tests among the participants, namely the word list learning test and the word list recall test from the Consortium to Establish a Register of Alzheimer’s Disease (CERAD), the verbal fluency test, and the digit-symbol substitution test (DSST). Kaplan et al. [25] analysed the effect of glucose, fat and protein intake on memory. Subjects consumed four test drinks containing placebo (water, lemon juice,sodium sacharin), carbohydrates (water, lemon juice, and dextrose monohydrate), protein (water, lemon juice, whey protein isolate) and fat (water, lemon juice, safflower oil emulsion). Fifteen and 60 minutes after consumption a paragraph recall test was performed immediately and after a delay. Results showed that, in the 15-minute test, ingestion of all three macronutrient drinks improved delayed recall and improved or tended to improve immediate recall compared to placebo. According to the authors, protein was the only macronutrient that slowed down forgetting, as intermediate and delayed results were almost identical in this group. Similar results were not observed 60 minutes after consumption. The differences in the conclusions drawn from the above studies suggest that the effect of proteins on memory and recall processes remains not fully understood, facilitating need for further research.

Fats are important macronutrients for the human body, as they constitute the basic building blocks of cell membranes and play a role in regulating the nervous system. These compounds perform many functions that enable the body to function properly and maintain homeostasis. Fatty acids constitute the primary source of energy for humans, provide thermal insulation, transport vitamins A, D, E and K, and are involved in the synthesis of steroid hormones, which are essential, among other things, for normal sexual development [26]. There are four classes of fats in the diet: saturated (SFA), trans, monounsaturated (MUFA) and polyunsaturated (PUFA). The summary of this information has been presented in Table 1.

**Table 1:** Summary of the influence of macronutrients on respective fields of cognitive function Arrows indicate the type of change (improvement or deterioration) based on studies.

Fats	Proteins	Carbohydrates
Polyunsaturated fatty acids:	↑ Visuospatial ability [22-24]	High-carbohydrate diet → Excess of calories:
↑ Information processing [20]	↑ Verbal fluency [22-24]	↓ Working memory [20]
↑ Episodic memory [5,36]	↑ Episodic memory [23]	↓ Attention [20]
↑ Language skills [36]	↑ Memory latency [25]	↓ Inflammation → ↑ Neuronal mortality [5,19]
↑ Visuospatial ability [36]	↓ Cognitive function [24]	Low-carbohydrate diet:
Saturated fatty acids:		↑ Anxiety [6]
↓ Prospective memory [20,37]		
↓ Learning [20,37]		
↓ Visuospatial ability [20,37]		
↓ Verbal fluency [20,37]		

Role of Saturated Fats/High-Fat Diet

A particularly crucial role is attributed to PUFAs such as omega-3 and omega-6, which may help to support cognitive functions like learning and memory. It has been suggested that polyunsaturated lipids, rich in omega-3 and omega-6 fatty acids, contribute to improved memory and faster processing of psychomotor information. Particular attention has been paid to omega-3 fatty acids, as they have shown to have a positive effect on cognitive functions in studies, i.e. improved spatial and semantic memory, learning, short-term memory and the recall process in both older and middle-aged individuals [20]. Similar observations were made by Charbit et al. [5], who confirmed the positive effect of omega-3 fatty acids on memory processes, particularly episodic memory, in a group of older adults with initially impaired cognitive function or metabolic disorders [5].

A meta-analysis by Shahinfar et al. [36] also highlighted the positive effects of omega-3 fatty acids on perceptual speed, language skills, episodic memory, visuospatial functions and overall cognitive performance. A dose-response study of omega-3 supplementation was also conducted, in which it was noted that the most consistent and pronounced effects were observed with a daily intake of approximately 1000-2500 mg. Importantly, no side effects were observed with supplementation.

Welty [27] focused primarily on the beneficial effects of one of the omega-3 fatty acids, specifically docosahexaenoic acid (DHA), which also contributes to the improvement of cognitive processes and the regulation of neuronal inflammation. These findings have been linked to the end products of DHA breakdown (D-series resolins, maresins and protectins), which are specialised lipid mediators that lead to the resolution of inflammation. DHA is also a precursor of neuroprotectin D1 (NPD1), which is responsible for increasing the expression of the peroxisome proliferator-activated receptor gamma (PPAR-γ) and amyloid precursor protein α, and for decreasing the expression of amyloid precursor protein β. This leads to a significant reduction in the release of Aβ42 and a reduction in Aβ peptide levels in neural tissue, resulting in a significant slowing of AD progression. It has also been demonstrated that individuals carrying the APOE4 gene exhibit reduced levels and uptake of DHA in the brain. Given all these mechanisms, supplementation and a high dietary intake of omega-3 fatty acids are particularly recommended for individuals with memory problems, a positive family history, and a predisposition to AD.

Du et al. [7] focused on analysing the impact of consuming high-fat cheese and cream on cognitive processes and dementia. It was found that a higher intake of high-fat dairy products was associated with a lower risk of dementia from any cause. This means that these individuals were less likely to develop AD, provided they were not carriers of the APOE4 gene. In contrast, a study by Park and Fulgoni [37] indicated that the consumption of full-fat dairy products was inversely proportional to cognitive function in older adults. The authors attributed this effect to the higher content of saturated fatty acids (SFAs). High SFA intake has also been linked to impaired memory processes, such as prospective memory, visuospatial learning and verbal memory. Similar effects were observed in the case of a diet rich in highly processed foods containing trans fats, the consumption of which was associated with a decline in visuospatial and verbal memory [20].

In summary, fats play a vital role in the body’s functioning, and their appropriate quantity and quality in the diet are important for maintaining health, including the proper functioning of the nervous system. However, it should be remembered that the type of fats consumed can affect not only metabolic and cardiovascular health, but also brain function and cognitive processes. 

Studies on Dietary Interventions

The available literature describes a range of clinical interventions undertaken to improve cognitive function, particularly in older adults, through lifestyle modification. However, most studies focus on a multidisciplinary approach, taking into account not only dietary changes but also increased physical activity, changes in the microbiota, and improved sleep. A second limitation arising from the study design was the selection of specific patient groups, e.g., those suffering from AD or diabetes.

Multifactorial interventions [8-11] demonstrated that, with an overall improvement in lifestyle, an increase in cognitive function as well as an improvement in memory is possible. Castillo-Bernad et al. [9] demonstrated that dietary modifications and exercise can significantly raise the overall cognitive level of participants; however, no improvement was observed in executive or visuoperceptual functions. Kurmi et al. [8], emphasised that only a combination of interventions yielded measurable results in improving the condition of patients with AD. They noted that physical activity was responsible for increased neuroplasticity, whilst an appropriate diet (Mediterranean, MIND or ketogenic) helped reduce inflammation in the body, thereby contributing to improved function of the nervous system. This effect can be enhanced by optimising the gut microbiota using prebiotics and probiotics. In turn, Givralli et al. [10] indicated that combining several strategies simultaneously may improve cognitive function and slow the progression of neurodegenerative diseases through the action of molecules such as brain-derived neurotrophic factor (BDNF). The authors noted, however, that the available studies approached the subject from many different perspectives and used varied diagnostic methods, which made it difficult to draw clear conclusions from the results obtained. These conclusions also correlated with the view of Rasmussen [11], who emphasised the importance of multidisciplinary intervention in delaying the progression of dementia, whilst remaining sceptical due to the continued lack of precise data. Before proceeding to a direct analysis of dietary interventions, it is worth mentioning the work carried out by Zhao et al. [38], who worked with UK Biobank statistical data, linking patients’ cognitive functioning to their socioeconomic status via the types of food they consumed on a daily basis. It was shown that the higher a person’s income, the greater the likelihood of sustained good cognitive function, with such individuals more frequently consuming products such as cheese, coffee or dried fruit.

When it comes to dietary changes suggested as optimal for delaying the progression of dementia, the Mediterranean diet or its correlation with the ‘Dietary Approaches to Stop Hypertension’ (DASH) diet in the form of the MIND diet are most frequently cited in the literature. Olmos et al. [29] demonstrated that after four months on the MIND diet, patients in the study group who were confirmed to have adhered correctly to the dietary recommendations performed significantly better on cognitive function tests at the end of the study (patients were assessed using the MMSE and CogniFit questionnaires). At the same time, elevated levels of BDNF were confirmed in these individuals’ urine, which the authors linked to increased potential for neuroplasticity. Furthermore, in their conclusion, the researchers highlighted the possible synergistic effect of combining regular physical activity with appropriate social support for individuals at risk of developing dementia, which correlated with earlier studies [8-11]. In a longer, three-year study, Dhana et al. [30] confirmed the findings of the previously discussed study [29], although the endpoints used there were cognitive test results corroborated by differences in the levels of amyloid-beta proteins and p-tau181. In another study [31], the collected data allowed for a link to be established between the Mediterranean diet and a reduction in plasma inflammatory markers such as IL-6, C-reactive protein, and lipopolysaccharide. At the same time, improvements in mitochondrial function and a reduction in oxidative stress were noted. These factors influence human metabolism in terms of carbohydrate metabolism, reducing the risk of the onset/progression of type 2 diabetes and lipid disorders, which improves the condition of the vascular system and slows the progression of neurodegeneration in older patients.

These correspond with the results obtained by Voigt et al. [32], where, after three years of following the MIND diet and engaging in regular physical activity, a reduction in patients’ body fat mass and a decrease in inflammatory protein levels in the blood were observed. In this case, the authors simultaneously examined the composition of the patients’ gut microbiota each year. Interestingly, changes occurred in both the group combining exercise with the diet and exercise alone; however, in the group following the MIND diet, this change occurred in a more stable manner, with the preservation of bacterial strains responsible for the synthesis of substances considered to have a beneficial effect on human cognitive function. Regarding the influence of the gut microbiota, it was noted that following a Mediterranean diet provided an optimal amount of fermentable fibre, which improved the biodiversity of the flora [3]. In conjunction with personalised probiotic and prebiotic therapy, a tightening of the intestinal barrier was observed, and consequently, a reduction in inflammatory markers. The patients reported reduced stress levels and, in general, improved mental functioning. The authors noted, however, that the interventions undertaken combined many elements, and the data should be interpreted as carrying a high risk of variability and may also differ between individuals.

Another type of intervention involved attempts to switch to a ketogenic diet. Xun et al. [33] indicated that inducing a state of ketosis in the body improved mitochondrial function and reduced insulin resistance, which in turn improved metabolic function, reducing neuronal disorders caused by systemic inflammation. In line with previous studies [4,15,31,32], a reduction in inflammation was associated with improved cognitive function. However, the authors themselves pointed to the small sample sizes of patient studies to date and the fact that most of the data originated from preclinical trials. Furthermore, as mentioned earlier [5], the duration of the intervention must always be taken into account, which in the case of the ketogenic diet may also be associated with negative psychological effects.

Although dietary interventions have been shown to be effective in halting or reducing cognitive decline in patients, it should be borne in mind that, unfortunately, cognitive changes cannot always be completely reversed. In a recent study using animal models [39], brain imaging and changes in protein levels (beta-III-tubulin, NeuN) demonstrated improvement following a dietary intervention (switch from a high-fat diet to a standard diet); however, it turned out that some of the processes associated with neuroinflammation were irreversible. This issue appears to be a new area for further research in the human population, but it does not constitute a contraindication to the use of interventions aimed, for example, at slowing down changes in the elderly. 

A brief summary of the conclusions from discussed studies is given in Figure 1.

**Figure 1:**
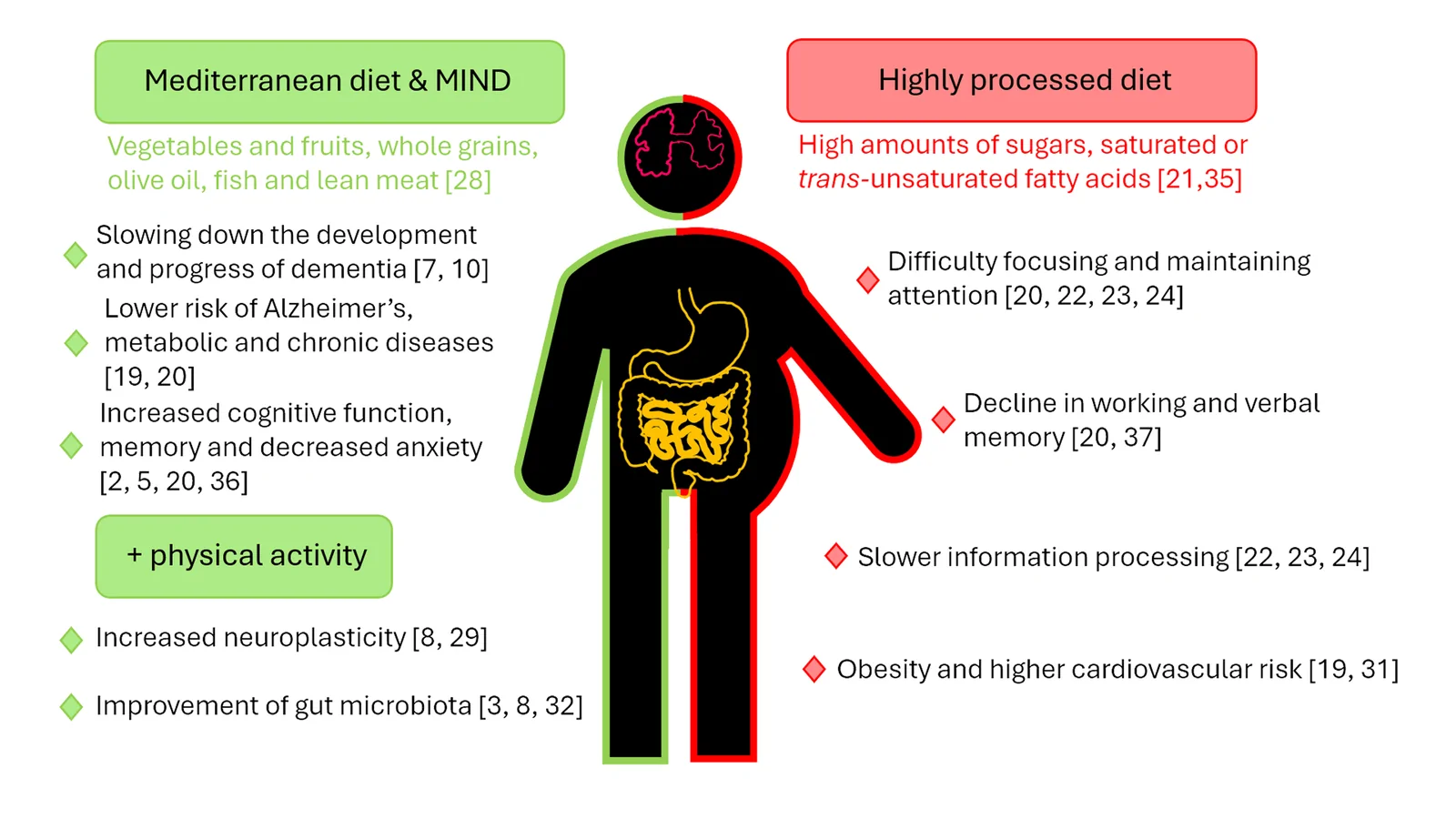
Summary of conclusions from the interventional research On the left is the data regarding the influence of dietary changes with addiction of physical activity and their effects on cognitive function (numbers in brackets indicate the appriate citations). On the right side is the data on effects of the consumption of highly processed diets on function of the central nervous system (CNS) [2,3,5,7,8,10,19-24,28,29,31,32,35-37]. Image credit: Created by the authors using MS PowerPoint (Microsoft Corp., Redmond, WA, USA) and Adobe Photoshop (Adobe Inc., San Jose, CA, USA).

Recommendations

In accordance with the recommendations of the Polish National Centre for Nutrition Education [40], the Mediterranean and MIND diets have also been identified as optimal dietary approaches aimed at reducing the risk of dementia. At the same time, to reduce the risk of cognitive decline, consuming foods such as green leafy vegetables, berries, nuts, whole grains, pulses, fish (not fried), poultry (not fried), oily sea fish is recommended, and using olive oil as the main fat used in meal preparation. At the same time, experts point out that the optimal approach is to replace animal fats with plant-based fats whenever possible.

## Conclusions

In summary, the available literature suggests that there is a correlation between the type of diet consumed, disturbances in the gut microbiome, and the occurrence of dementia and cognitive decline. Despite the demonstrated multifactorial nature of this phenomenon, it appears that the greatest role in this regard is played by a high proportion of fats and diets conducive to the development of obesity, such as those containing large amounts of carbohydrates. In the immediate future, studies focusing strictly on the human population but using single variables, rather than multifaceted interventions, appear to be most useful in analysing this issue, as they could identify the most significant elements that could be introduced into everyday clinical practice.
